# Metabolic syndrome and its correlated factors in an urban population in South West of Iran

**DOI:** 10.1186/2251-6581-12-11

**Published:** 2013-02-08

**Authors:** Hajieh Shahbazian, Seyed Mahmoud Latifi, Mohammad Taha Jalali, Heshmatollah Shahbazian, Reza Amani, Abdolrasool Nikhoo, Armaghan Moravej Aleali

**Affiliations:** 1 Diabetes Research Center, Ahvaz Jundishapur University of Medical Sciences, Ahvaz, 61157-15794 Iran; 2 Nutrition Research Center, Ahvaz Jundishapur University of Medical Sciences, Ahvaz, Iran; 3 Infection Disease Department, School of Medicine, Ahvaz Jundishapur University of Medical Sciences, Ahvaz, Iran

**Keywords:** Metabolic syndrome, Hypertension, Hypertriglyceridemia, Low HDL, Abdominal circumference, Blood glucose

## Abstract

**Background:**

This study was designed to assess the prevalence of metabolic syndrome and its correlated factors in an urban population in Ahvaz.

**Methods:**

This descriptive analytical study performed with random cluster sampling method in 6 health centers in Ahvaz. In each selected center, 55 households were randomly selected. A questionnaire included: age, sex, marital status, ethnicity, education level, family history of diabetes (DM), Hypertension (HTN) and obesity, smoking and parity and previous history of gestational diabetes Mellitus in women were filled for each person.

Blood pressure, weight, height, body mass index (BMI), abdominal and waist circumference were measured in each participant. Fasting blood glucose (FBS), serum total cholesterol, triglyceride and high density lipoprotein (HDL) level were measured in fasting blood sample.

**Results:**

The mean age of all participants was 42.27 ± 14 years (44.2 ± 14.26 years in men and 40.5 ± 13.5 in women). From total 912 participant, 434(47.2%) were men and 478(52.8%) women. Prevalence of metabolic syndrome based on ATPIII criteria (update2005) was 22.8% (15.9% in men and 29.1% in women) that showed significant difference (P = 0.0001). Prevalence of each component of MS in studied population was: 29.4% for abdominal obesity, 40.7% for high TG level, 40.2% for low HDL, 15.4% for hypertension and 37.8% for abnormal FBS. Among these factor, age of patients, BMI, sex had significant differences between persons with or without Ms (P = 0.0001). Ethnicity (Arab or Persian), cigarette smoking and family history of diabetes mellitus, hypertension and obesity, marital statues, education level, parity and previous history of GDM in women showed no significant differences between persons with MS and without MS.

**Conclusion:**

Metabolic syndrome has high prevalence in our population and its prevalence increases with increasing age and BMI. Women are at higher risk for metabolic syndrome than men.

**Electronic supplementary material:**

The online version of this article (doi:10.1186/2251-6581-12-11) contains supplementary material, which is available to authorized users.

## Introduction

Metabolic syndrome (MS) or X syndrome refers to simultaneous occurrence of cardiovascular risk factors or type 2 diabetes such as abdominal obesity, high blood pressure and abnormal carbohydrate and lipid metabolism (hypertriglyceridemia, elevated blood glucose) and decrease in high density lipoprotein (HDL) level [1]. Although there are various definitions for metabolic syndrome, but the most practical method in clinical diagnosis is using Adult treatment Panel III (ATPIII). According to this definition the person must have at least three factors of cardiovascular risk factors at the same time. Studies have shown that there is a coincidence of metabolic factors in different individuals, and coexistence of these factors is more harmful than one of them [2]. As a matter of fact metabolic syndrome is like a bridge between diabetes and cardiovascular disease [1]. About 50% of patients with type 2 diabetes are suffering from metabolic syndrome and these people have more chances for stroke, retinopathy, neuropathy and microalbminuria [1]. Studies have shown that more than half people with Acute Coronary syndrome have three or more Components of metabolic syndrome [3]. High prevalence of metabolic syndrome (95%) has been reported in patients with Peripheral Arterial Disease [4]. Prevalence of metabolic syndrome is increasing in different region like Asia [5] and developing countries [6]. Prevalence of metabolic syndrome has been reported between 12.8% to 41.1% in different part of the world [7]. In DECODE study that was conducted in 9 European countries, MS was detected in 32% of men and 28.5% of women [8]. Prevalence of MS was reported in recent studies in Estonia 25.9% [9], Norway 25.9% [10], turkey 28/8% [11] and luxamburg 24/7% [12].

Studies in different provinces in Iran showed that the prevalence of metabolic syndrome was between 21.9% to 31.1% [1, 2, 13, 14]. Many factors including: age, race, weight, menopause in women, smoking, low income economies, high carbohydrate intake, no alcohol consumption, low physical activity [15], consumption of soft drink [16], antipsychotic drugs [17] poor cardiovascular fitness [18] and Genetic factors may play a role in metabolic syndrome. However, early identification of patients and treatment with appropriate medical and educational programs can be an effective step in control and reduce the incidence of metabolic syndrome and also cardiovascular disease and diabetes [1]. This study was designed to assess the prevalence of MS and its risk factors in an urban population in Ahvaz (South West of Iran).

## Materials and methods

This descriptive analytical study performed with random cluster sampling method in 6 health center in Ahvaz. In each selected center, 55 households were randomly selected. After obtaining informed consent by volunteers, they were invited to participate in this study.

A questionnaire included: age, sex, marital status, ethnicity, education level, family history of diabetes (DM), Hypertension (HTN) and obesity, smoking and parity and previous history of gestational diabetes Mellitus in women were filled for each person. Blood pressure, weight, height, body mass index (BMI) [Weigh(kg)/Height(m)^2^], abdominal and waist circumference were measured in each participant. Blood pressure was measured by a standard sphygmomanometer after 15 minutes rest in a sitting position. The cuff was placed on the right arm at the heart level and then quickly pushes the device until 30 mm Hg above radial pulse disappearance. Blood pressure was measured twice at least 30 minutes interval between two measurement and mean of these two measurements, was taken as blood pressure. Anthropometric measurements were taken after removing shoes and wearing a light dress. Weight and height were measured according to the standard program. Waist circumference was measured at the midpoint between the lowest rib and the upper lateral border of the right iliac crest and hip circumference at the point of maximum hip diameter.

After 12 h of fasting, blood samples were taken in the morning. Samples was centrifuged, serum stored in the refrigerator and was sent to Diabetes Research Center laboratory.

Triglyceride (TG), Fasting Blood Sugar (FBS), Cholesterol and high density lipoprotein (HDL) were measured using an enzymatic colorimetric method with Pars Azmoon kit. (With Biotechnical instruments model BT-3000 Germany).

For diagnosis of metabolic syndrome at least three of the following five components were considered necessary (according to ATP III criteria update 2005) [19, 20].

1. Abdominal obesity (Waist circumference ≥ 102 cm in men and ≥ 88 cm in women).
2. TG ≥ 150 mg/dl or history of drug consumption for hypertriglyceridemia.
3. HDL ≤ 40 mg/dl in men and ≤50 mg/dl in women or history of drug consumption.
4. BP Siastolic ≥ 130 mmhg or BP diastolic ≥ 85 mmhg or history of anti hypertensive drug consumption.
5. FBS ≥100 mg/dl, history of diabetes mellitus history or using anti diabetic drugs.

Sample size was calculated as 912 and EPI2000 descriptive statistics was used to provide figures and tables. Chi-square test, logistic regression and trend test was used for correlation assessment SPSS software 19 and EPI2000 descriptive statistics was used to provide figures and tables. P = 0 < 0.05 was considered as significant.

## Results

From total 912 participant, 434 (47.2%) were men and 478(52.8%) women. The mean age of all participants was 42.27 ± 14 years (44.2 ± 14.26 years in men and 40.5 ± 13.5 in women). Prevalence of metabolic syndrome based on ATPIII criteria (update2005) was 22.8% (15.9% in men and 29.1% in women) that showed significant difference (P = 0.0001).

Trend test showed increased prevalence of metabolic syndrome with increasing age in total sample and both sexes. (P = 0.0001) (Table 1).

**Table 1:** **Prevalence of metabolic syndrome according to sex and age group**

All population studies				
Age (year)	N	No. of MS (%)	Confidence Interval (CI)	Odd ratio (OR)
**20-29**	203	13(6.4)	3.59-10.94	1
**30-39**	198	29(14.6)	10.14-20.47	2.51
**40-49**	226	61(27)	21.43-33.37	5.40
**50-59**	176	59(33.5)	26.68-41.05	7.37
**60-69**	80	34(42.5)	31.68-54.05	10.8
**≤ 70**	29	12(41.4)	24.8-60.89	1.32
**Total**	912	208(22.8)		
	**Men**			
**20-29**	87	5(5.7)	2.11-13.44	1
**30-39**	78	11(14.1)	7.58-24.25	2.69
**40-49**	112	20(17.9)	11.54-26.52	3.57
**50-59**	94	16(17)	13.2-26.45	3.36
**60-69**	44	11(25)	13.7-40.65	5.47
**≤ 70**	19	6(31.6)	13.57-56.52	7.57
**Total**	434	69(15.9)		
	**Women**			
**20-29**	116	8(6.9)	3.24-13.56	1
**30-39**	120	18(15)	9.37-22.94	2.38
**40-49**	114	41(36)	27.38-45.48	7.58
**50-59**	82	43(52.4)	41.14-63.43	14.88
**60-69**	36	23(63.9)	46.22-78.67	23.88
**≤ 70**	10	6(60)	27.37-86.31	20.25
**Total**	478	139(29.1)		

**Figure 1:**
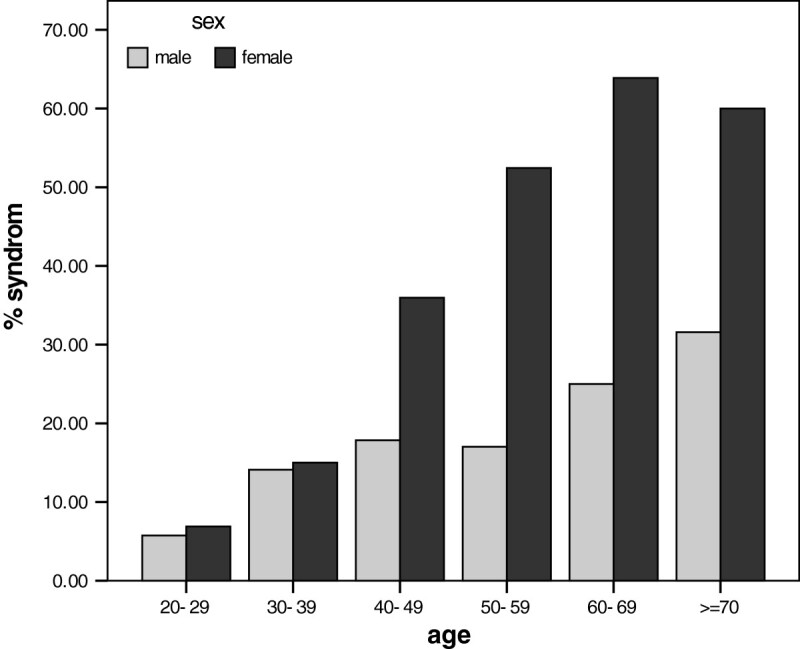
**Prevalence of Metabolic syndrome in different sex and age group.**

Figure 1 shows the prevalence of MS in different age group in both sexes.

The highest prevalence of metabolic syndrome was 42.5% that was seen in age group 60–69 years (31.6% in men in with ≥70 years, 63.9% in women with 60–69 years). Prevalence of each component of MS in studied population was: 29.4% for abdominal obesity, 40.7% for high TG level, 40.2% for low HDL, 15.4% for hypertension and 37.8% for abnormal FBS (15.3% DM and 22.6% impaired fasting glucose (IFG).

Abdominal obesity showed highest prevalence in age group 50–59 years (39.1%). High TG was more prevalent in 50–59 years group (52.2%) and Low HDL in 30–39 years (47.3%). The highest prevalence of high blood pressure and abnormal FBS were seen in age group ≥ 70 years (50% and 62.1% respectively) Prevalence of each component of MS in both sexes in each age group was shown in Table 2.

**Table 2:** **Prevalence of each MS component according to age and sex**

Total population					
Age(year)	Abdominal obesity (%)	TG ≥ 150 (%)	HDL ≤ 40,50 (%)	BP ≥ 130/85 (%)	FBS ≥ 100 (%)
**20-29**	13	22.9	42	0.5	17.1
**30-39**	30.3	35.5	47.3	6.5	22
**40-49**	32.9	48.2	41.1	12.2	45.7
**50-59**	39.1	52.2	32.8	31.5	55.3
**60-69**	35.8	51.3	33.3	35.8	60
**≤ 70**	33.3	41.4	36.7	50	62.1
**Total**	29.4	40.7	40.2	15.4	37.8
**Men**					
**20-29**	10.2	32.2	30.7	0	21.8
**30-39**	18.8	45.6	38.8	7.5	27.8
**40-49**	14.2	53.6	23	9.7	47.3
**50-49**	16.8	56.4	25.3	23.2	52.6
**60-69**	11.4	54.5	22.7	25	56.8
**≤ 70**	21.1	36.8	36.8	52.6	47.4
**Total**	15	47.8	28.5	13.7	40.8
**Women**					
**20-29**	15.1	16.1	50.4	0.8	13.6
**30-39**	38	28.9	52.9	5.8	18.2
**40-49**	50.8	43.1	58.5	14.7	44.1
**50-49**	64.3	47.6	41.2	41	58.3
**60-69**	64.9	47.2	45.9	48.6	63.9
**≤ 70**	54.5	50	36.4	45.5	90
**Total**	42.4	34.2	50.7	16.9	35.1

In total subjects, 22% were normal (no any component of MS) 29.9% had one component, 26.1% two, 13.8% three, 6.9% four and 1.4% have five component of metabolic syndrome (Table 3).

**Table 3:** **Number of metabolic syndrome component in studied population**

No. of component	No.	%
**No any component**	200	22
**1 component**	272	29.9
**2 component**	238	26.1
**3 component**	126	13.8
**4 component**	63	6.9
**5 component**	13	1.4
**Total**	912	100

The prevalence of abdominal obesity was 15% in men and 42.4% in women that showed significant difference (P = 0.0001). The prevalence of hypertriglyceridemia was higher in men (47.8%) than women (34.2%) with significant difference (P = 0.0001). Low HDL was detected in 50.7% of women and 28.5% of men with significant difference (P = 0.0001). Rate of high blood pressure (BP ≥ 130/85) in men was 13.7% and in women 16.9% with no significant difference (P = 0.17). Prevalence of high FBS was 40.8% in men and 35.1% in women that showed significant difference (P = 0.041). Factors that can affect the incidence of MS are seen in Table 4.

**Table 4:** **Logistic regression model**

	B	S.E	P value	OR	Confidence Interval(CI)
**Sex**	0.73	0.24	0.00	2.08	1.30-3.33
**Marriage status**	-0.32	0.46	0.47	0.72	0.29-1.78
**Ethnic**	-0.36	0.24	0.13	0.69	0.43-1.11
**Smoking**	0.52	0.38	0.17	1.69	0.79-3.61
**Family history**					
**Overweight**	0.44	0.23	0.05	1.56	0.99-2.45
**Diabetes**	-0.08	0.23	0.71	0.91	0.57-1.45
**HTN**	0.14	0.22	0.52	1.15	0.74-1.80
**BMI**					
**BMI ≤ 19**				1	
**19.1-24.99**	0.25	1.11	0.81	1.29	0.14-11.39
**25-29.99**	1.00	1.10	0.36	2.73	0.31-23.80
**≥30**	2.34	1.10	0.03	10.42	1.18-91.40
**Age**					
**20-29**				1	
**30-39**	0.38	0.44	0.37	1.47	0.62-3.49
**40-49**	1.57	0.44	0.00	4.82	2.02-11.48
**50-59**	1.90	0.45	0.00	6.69	2.75-16.30
**60-69**	2.58	0.52	0.00	13.19	4.74-36.70
**≥70**	2.25	0.70	0.01	9.57	2.39-38.20
**Education level**					
**Non educated**				1	
**Primary**	0.02	0.33	0.93	1.03	0.53-1.98
**High school**	-0.54	0.33	0.10	0.58	0.30-1.12
**University**	-0.42	0.42	0.31	0.65	0.28-1.50

Among these factor, age of patients, BMI, sex, had significant differences between persons with or without MS (P = 0.0001). Ethnicity (Arab or Persian), cigarette smoking and family history of diabetes, hypertension and obesity, marital status, level of education, and parity number and previous history of GDM showed no significant differences between persons with MS and without MS (P > 0.05).

## Discussion

The metabolic syndrome is an important risk factors for diabetes type 2 and cardiovascular diseases, thus the clinical implication of diagnosis of MS is identification of patients who needs aggressive life style modification [21–23].

The results of this study showed that the prevalence of metabolic syndrome is high in studied population and it increases with increment of age and BMI significantly. The prevalence of MS was 22.8% (15.9% in men 29.4% in women) with significant difference. Smoking, ethnicity, level of education, family history of diabetes, hypertension and obesity, marital status, and parity and history of GDM in women had no effects on prevalence of MS.

The prevalence of MS in this study is similar to studies in Zanjan 23% [24] and Zahedan 21% [25] but lower than some other studies in Iran. MS was detected in 32% of population in Yazd [2] and about 30% in Tehran [13, 14]. One reason for these differences may be heterogeneity of studied population. In this study, only about 31% of the population was in age group higher than 50 years, but in Yazd [2] study more than 50% of population was in this high risk age group. Given the increasing prevalence of metabolic syndrome with increasing age, this can be one reason for lower prevalence of MS in our study. Genetic factors, race, nutrition and physical activity can also have an impact on the prevalence of MS. Differences in prevalence of metabolic syndrome in communities are because of differences in prevalence of each component of MS. For example, diabetes prevalence in this study was 15.3% but it was 25.7% in Tehran [13] study.

Prevalence of low HDL in our study was 40.2%, but in Tehran study it was 63% [13], in Yazd [2] 35% in men and 64% in women and in Zanjan [24] 93% in women and 63% in men.

Genetic factors are important in the incidence of low HDL. Existence of specific genes in addition to nutrition and physical activity and smoking can have effect on the prevalence of low HDL [26–30]. The prevalence of MS has been reported in other parts of the world with different diagnostic criteria. In a study in USA 34.5% of people had MS based on ATPIII 2002 criteria [31].

In DECODE study that was conducted in 9 European countries, MS was detected in 32% of men and 28.5% of women [8]. High prevalence of MS was reported in recent studies in Estonia 25.9% [9], Norway 25.9% [10], Turkey 28/8% [11] and luxamburg 24/7% [12].

In a recent study in Korea [32] MS was seen in 6.4% of adolescences and 22.3% of adults.

Many factors including: age, race, weight, menopause in women, smoking, low income economies, high carbohydrate intake, no alcohol consumption, low physical activity [15], consumption of soft drink [16], antipsychotic drugs [17], poor cardiovascular fitness [18] and Genetic factors may play a role in metabolic syndrome.

A parental history of metabolic syndrome increases the risk, and genetic factors may account for as much as 50% of the variable in level of metabolic syndrome traits in offspring [33–36]. In most studies (2, 8.12) increasing age was the key factor affecting the prevalence of metabolic syndrome and it also showed in our study. Increasing in BMI was correlate with increasing prevalence of MS in this study. This is in agreement with other studies [37]. There is controversy about the relation between sex and MS in different studies. In this study, prevalence of metabolic syndrome was significantly higher in women than men. This is in agreement with other studies (2, 8, 14, 37). But in some studies, the prevalence of MS in men is more than women [38]. That’s may be become of reduction of physical activity in Iranian women than may cause higher rate of abdominal obesity and low HDL. Genetic, cultural, physical activity and nutritional differences can be the cause of controversies. This association was seen in some other studies [39, 40]. In this study, smoking, ethnicity (Arabs and Persians) and marital status and education does not affect the rate of metabolic syndrome. Smoking more than 20 cigarettes per day has been associated with increased risk of metabolic syndrome in some studies [41, 42].

Strengths of this study are that it is a population based study in an urban population of Iran, extensive information of confounders and a relatively large sample size. One limitation was that, this study was cross sectional that does not allow us to draw any causal interference. Therefore in the future large prospective studies should be used to confirm the association between above mentioned factors and metabolic syndrome.

## Conclusions

Metabolic syndrome has high prevalence in our population and its prevalence increases with increasing age and BMI. Women are at higher risk for MS than men.

## Authors’ original submitted files for images

Below are the links to the authors’ original submitted files for images. Authors’ original file for figure 1
