# Supplementary figures and images for: Metabolic syndrome and its correlated factors in an urban population in South West of Iran
Source: J Diabetes Metab Disord. 2013 Feb 8;12:11. doi: 10.1186/2251-6581-12-11 (PMC3598198; doi:10.1186/2251-6581-12-11)

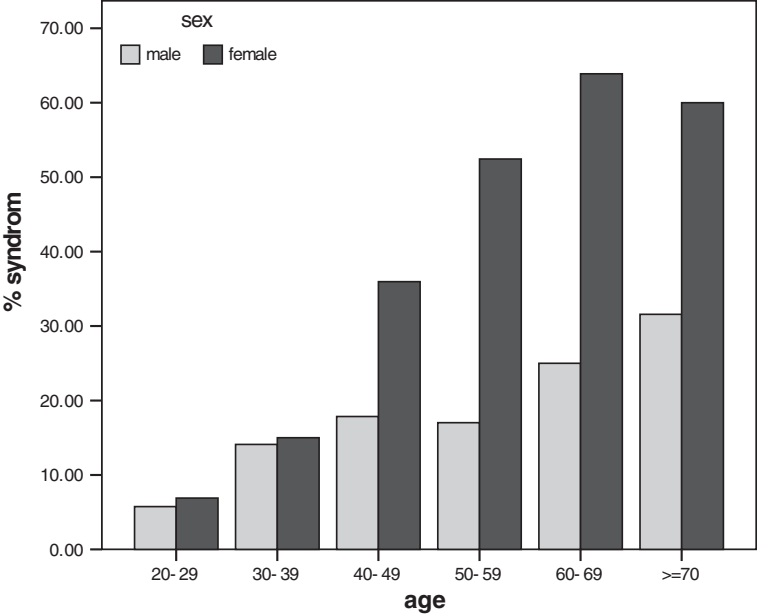

Supplement: Supplementary file 1 — Authors’ original file for figure 1 [file 40200_2012_35_MOESM1_ESM.pdf]
